# Adsorption Removal of Mo(VI) from an Aqueous Solution by Alumina with the Subsequent Regeneration of the Adsorbent

**DOI:** 10.3390/ijms24108700

**Published:** 2023-05-12

**Authors:** Alexandra Yu. Kurmysheva, Marina D. Vedenyapina, Stanislav A. Kulaishin, Pavel Podrabinnik, Nestor Washington Solís Pinargote, Anton Smirnov, Alexander S. Metel, José F. Bartolomé, Sergey N. Grigoriev

**Affiliations:** 1 Laboratory of Electric Current Assisted Sintering Technologies, Moscow State University of Technology “STANKIN”, Vadkovsky per. 1, 127055 Moscow, Russia; p.podrabinnik@stankin.ru (P.P.); nw.solis@stankin.ru (N.W.S.P.); a.smirnov@stankin.ru (A.S.); s.grigoriev@stankin.ru (S.N.G.); 2 N. D. Zelinsky Institute of Organic Chemistry, Russian Academy of Sciences, Leninsky Prospect 47, 119991 Moscow, Russia; mvedenyapina@yandex.ru (M.D.V.); s.kulaishin@mail.ru (S.A.K.); 3 Department of High-Efficiency Machining Technologies, Moscow State University of Technology “STANKIN”, Vadkovsky per. 1, 127055 Moscow, Russia; a.metel@stankin.ru; 4 Instituto de Ciencia de Materiales de Madrid (ICMM), Consejo Superior de Investigaciones Científicas (CSIC), c/Sor Juna Inés de la Cruz, 3, Cantoblanco, 28049 Madrid, Spain; jbartolo@icmm.csic.es

**Keywords:** molybdenum, wastewater treatment, molybdate, adsorption, aluminum oxide, adsorbent regeneration, desorption

## Abstract

Industrial wastewater is the main source of an excessive amount of molybdenum (Mo) in natural ecosystems. It is necessary to remove Mo from wastewater before it is discharged into the environment. Molybdate ion(VI) is the most common form of Mo in natural reservoirs and industrial wastewater. In this work, the sorption removal of Mo(VI) from an aqueous medium was evaluated using aluminum oxide. The influence of such factors as the pH of the solution and the temperature was evaluated. Three adsorption isotherms, namely, Langmuir, Freundlich and Temkin, were used to describe the experimental results. It was found that the pseudo-first order kinetic model better fits the kinetic data of the adsorption process, and the maximum Mo(VI) adsorption capacity was 31 mg/g at 25 °C and pH 4. The thermodynamic parameters indicated that the process of Mo(VI) adsorption on Al_2_O_3_ was exothermic and spontaneous. It was shown that the adsorption of Mo strongly depends on pH. The most effective adsorption was observed at pH values below 7. Experiments on adsorbent regeneration showed that Mo(VI) can be effectively desorbed from the aluminum oxide surface into a phosphate solution in a wide range of pH values. After the desorption of Mo(VI) in a phosphate solution, alumina was found to be suitable for repeating the procedure at least five times.

## 1. Introduction

Molybdenum (Mo) is an essential microelement for humans, animals and plant life [1]. It is also widely used for different industrial applications such as high-speed tools, coating processes and metallurgy for improving alloys [2,3,4,5]. For adults and older children, the recommended dietary intake is 75–250 μg/day [6]. However, as for all elements, high doses of Mo can be detrimental to plant and animal health, including human health, and may lead to gastrointestinal disorders, growth retardation, anemia, hypothyroidism, bone and joint deformation, infertility, impaired liver and kidney function, and death [2,7,8]. The average concentration of Mo in natural water reservoirs is normally 10 μg/L or less, but cases of significant excesses of this concentration (up to mg/L) are quite common [8]. The elevated Mo content in ecosystems is the result of anthropogenic activities such as mining and industrial activities associated with the production of alloys, catalysts, ceramics, lubricants and pigments [1,8]. In natural water reservoirs and industrial wastewater, Mo exists mainly in the form of Mo(VI) molybdate oxoanions. At most pH values, the dominant form of Mo(VI) is MoO_4_^2−^ [9].

Considering the value of Mo for industry and the fact that its penetration into the natural environment can harm the life of ecosystems, it is critical to remove MoO_4_^2−^ from industrial wastewater. Today, several methods for removing Mo have been developed such as chemical deposition [10], ion exchange [11] and adsorption [7]. The latter approach became the most widespread due to its high efficiency, low cost and flexibility in application [7]. Many studies are devoted to the adsorption recovery of Mo(VI) with various adsorbents, including activated carbon [12], ion exchange resin [13], silica-based adsorbent [14], a metal–organic framework [15] and biochar [16]. Aluminum oxide is one of the most available adsorbents for extracting Mo(VI) from solutions. The application of alumina for this purpose has been observed in other works [17,18,19]. Furthermore, it was found that the adsorption capacity of soils with respect to Mo(VI) increases with the amount of alumina it contains [20]. Being available, inert to aggressive media, nontoxic and easy to operate, alumina is considered to have good potential for use as an adsorbent for removing Mo(VI) [21,22].

Adsorbate desorption is a crucial step in evaluating the efficiency of adsorbent regeneration. The authors of [23] showed the applicability of a NaOH solution for the efficient desorption of Mo(VI) from the surface of drinking water treatment residues (DWTRs)—a by-product containing higher Al and Fe contents. Another iron-containing adsorbent, ZnFe_2_O_4_, was also effectively regenerated in a NaOH solution in [24]. A similar result was obtained by the authors of [25], in which the sulfuric-acid-modified cinder adsorbent was regenerated from molybdate ions in an alkaline medium. In [26], various soil samples saturated with Mo(VI) were treated with a phosphate solution, which led to the desorption of molybdate from their surface. In addition, the presence of phosphate ions in an aqueous solution significantly suppresses the adsorption of molybdate [6,9,27] regardless of the medium pH. However, the process of the desorption of Mo(VI) from the aluminum oxide surface has not received sufficient attention.

In this work, the adsorption of Mo(VI) was investigated depending on the pH and temperature of commercial alumina. The kinetics and thermodynamics of Mo(VI) sorption were studied. To determine the effectiveness and expediency of using Al_2_O_3_ as an adsorbent for the removal of Mo(VI) from aqueous solutions, the aluminum oxide was regenerated in an alkaline and phosphate medium.

## 2. Results and Discussion

### 2.1. Characterization of Adsorbent

**Figure 1:**
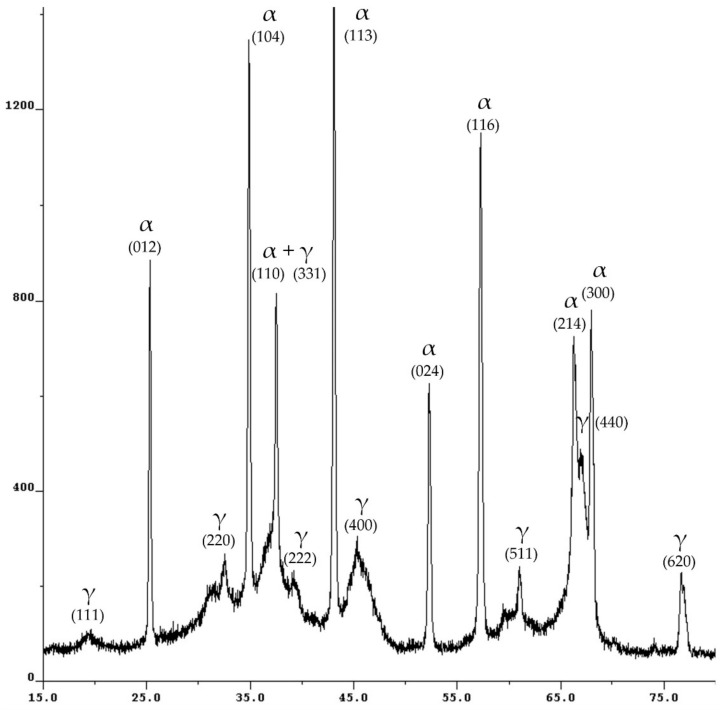
The XRD pattern of the alumina specimen.

The XRD analysis showed that the specimen contains two crystalline phases: α-Al_2_O_3_ and γ-Al_2_O_3_. It can be seen from the XRD pattern (Figure 1) that alumina (α-Al_2_O_3_) has a high degree of crystallinity, while the broad diffraction peaks of γ-Al_2_O_3_ indicate the opposite. In addition, broad diffusion peaks suggest the presence of an amorphous phase. After the quantitative analysis of the XRD patterns, it was found that the commercial specimen of the aluminum oxide contains a 15.7% mass of the α-Al_2_O_3_, a 39.0% mass of the γ-Al_2_O_3_ and a 45.3% mass of an amorphous phase [28,29].

According to the Brunauer–Emmett–Teller (BET) test results, Al_2_O_3_ is a microporous sample with an average diameter and a total pore volume of 0.017 nm and 8 × 10^−5^ m^3^/g, respectively. The specific surface area (S_BET_) was 65.5 m^2^/g.

The pH of the point of zero charge (pH_PZH_) is a pH at which an equal amount of positive and negative charges is observed on the surface of a substance [30]. If pH is lower than the pH_PZH_, an adsorbent has a positive surface charge, whereas preferentially negative ions are adsorbed (anions). On the contrary, cations are collected on the surface if pH is higher than the pH_PZH_. In this work, the pH_PZH_ was measured by the potentiometric titration method (see Section 3). Before titration, a small volume of a 1 M HNO_3_ solution was added to the suspension to protonate the surface areas. Then, the suspension was titrated by adding 0.05 mL of NaOH (0.1 M) accompanied with constant mixing.

**Figure 2:**
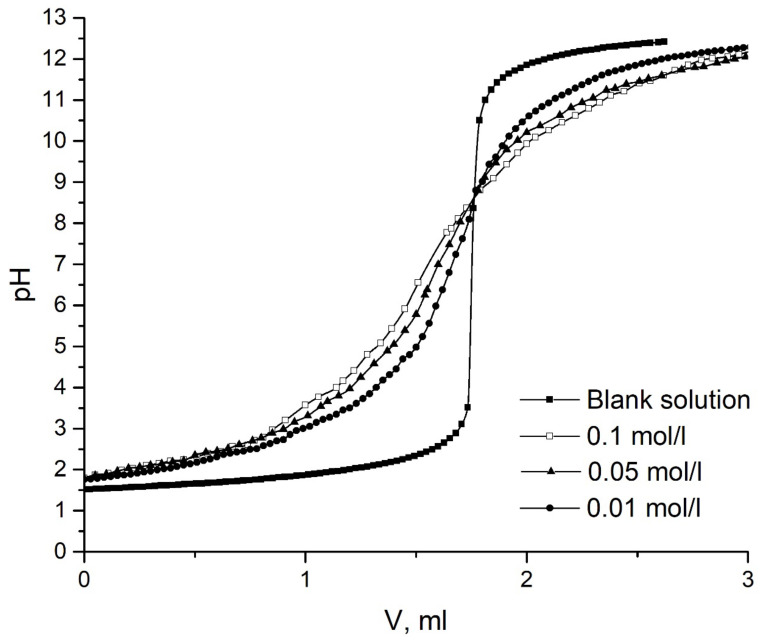
The curves of potentiometric titration by 0.1 M NaOH solution of NaNO_3_ solution and Al_2_O_3_ suspension (g/L) by 0.1 M NaOH solution at various concentrations of the supporting electrolyte, mol/L.

The pH of the pH_PZC_ of the adsorbent was found to be 8.8 (Figure 2). This indicates that the surface of Al_2_O_3_ will be positively charged at a pH below 8.8 and negatively charged at a pH above 8.8.

### 2.2. Sorption Studies

#### 2.2.1. Effect of pH

**Figure 3:**
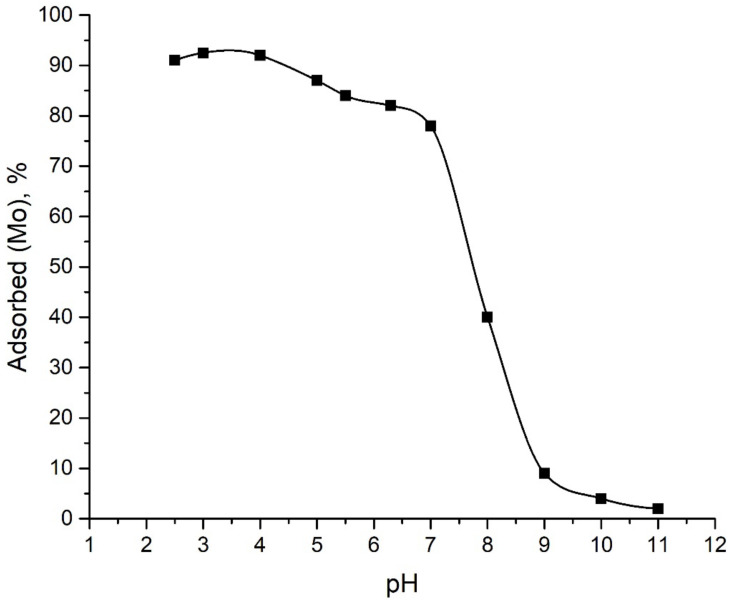
Adsorption of Mo onto Al_2_O_3_ under different pH.

Figure 3 shows the dependence of Mo(VI) adsorption on Al_2_O_3_ in the pH range of 2.5–11. The efficiency of Mo(VI) removal from the solutions was more than 90% at pH levels from 2.5 to 4 and then decreased with increasing pH. At pH 11, only about 2% of molybdate ions from the solution were adsorbed on alumina. A similar trend for Mo(VI) adsorption was observed on several different adsorbents [24,31,32].

The pH of the solution and the surface charge of the sorbent are critical factors in the process of Mo(VI) adsorption. According to [33,34], molybdate anion MoO_4_^2−^ is the dominating form of Mo at pH 5–6. Under more acidic conditions, molybdate is protonated to less-charged anions (HMoO^4−^), and in strongly acidic environments, neutral molybdic acid MoO_3_(H_2_O)_3_ appears. Furthermore, molybdate is capable of forming isopolymetallates such as Mo_7_O_2_^46−^ or Mo_8_O_2_^64−^ at pH levels below 5–6. However, polynuclear forms are found only at relatively high concentrations of Mo at 10^−4^ mol/kg of water or higher [8]. In the case of natural waters, pH is normally >5, and the concentration of Mo is several orders lower than that necessary for forming polynuclear particles [8]. Thus, the molybdate ion is anticipated to be a predominant substance in solutions in all the natural reservoirs.

The surface charge of the adsorbent undergoing protonation/deprotonation depends on the pH of the solution. Taking into account the pH value of the point of zero charge for the Al_2_O_3_ sample—8.8—the total surface charge at pH < 8.8 is positive, which is favorable for the adsorption of anions. This explains the high absorption of Mo ions in the pH range from 2.5 to 7. With a further increase in pH and as a result of the accumulation of a negative charge on the aluminum oxide surface and an increase in repulsive forces, the adsorption of Mo ions decreased.

These results show that the pH value plays an important role in the sorption process. A change in pH controls the sign and magnitude of the charge both on the surface of the adsorbent and on metal ions in the solution, which, consequently, affected the efficiency of Mo(VI) adsorption. Therefore, based on the results obtained, for further sorption studies, a pH value was preserved at value 4.

#### 2.2.2. Adsorption Kinetics

**Figure 4:**
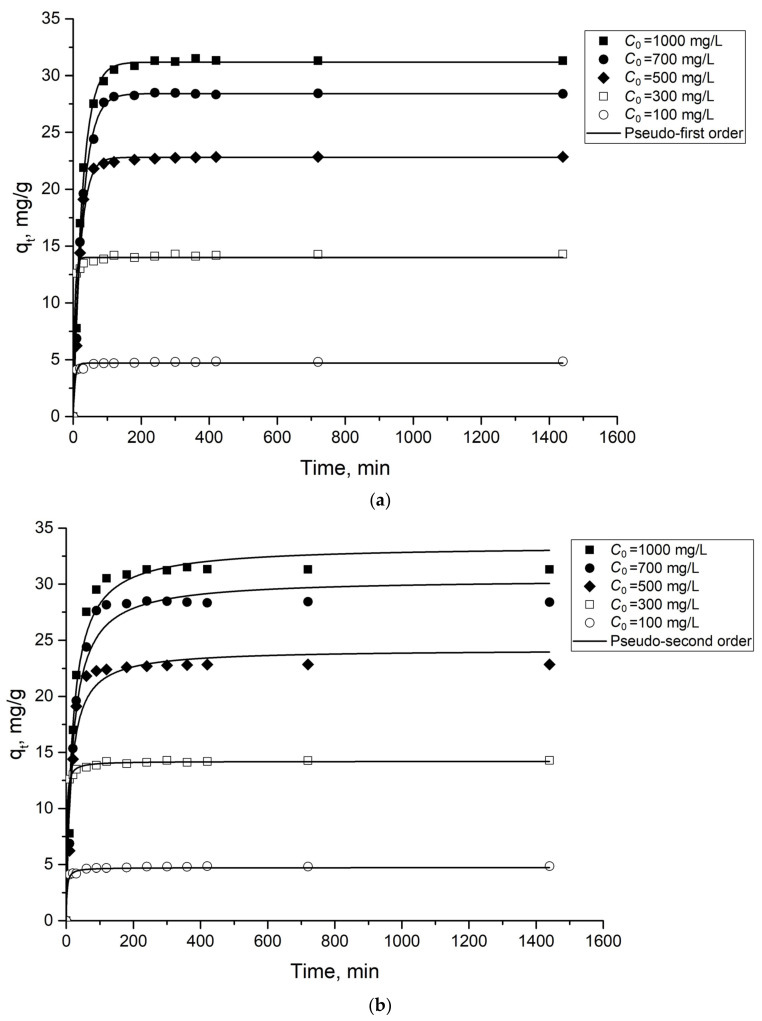
Pseudo-first (**a**) and Pseudo-second (**b**) order kinetic model of molybdate adsorption onto the studied material. Dots are experimentally obtained data for various *C*_0_; lines are the results of model calculations.

Adsorption kinetics is rather important for assessing the efficiency of an adsorbent. The kinetic curves of molybdate adsorption with Al_2_O_3_ sorbent are shown in Figure 4. The plot demonstrates that adsorption equilibrium is achieved rapidly (less than 100 min). To describe the kinetic curve, four kinetic models were used: a pseudo-first order (PFO) kinetic model [35], a pseudo-second order kinetic model (PSO) [36], the intraparticle diffusion model (IPD) [37] and the Elovich model [38].

Equations (1) and (2) describe the kinetic models of PFO and PSO, respectively:

(1) $$q_{t}=q_{e}\cdot \left(1-{exp}^{-k_{1\cdot }t}\right),$$

(2) $$q_{t}=\frac{q_{e}^{2}\cdot k_{2}\cdot t}{1+q_{e}^{2}\cdot k_{2}\cdot t},$$

where $k_{1}$(min^−1^) and $k_{2}$ (g/(mg∙min)) are velocity constants, and $q_{e}$ and $q_{t}$ (mg/g) are the amount of adsorbed ions of molybdenum at equilibrium and at time t, respectively.

Equations (3) and (4) describe IPD and Elovich model, respectively:

(3) $$q_{t}=k_{pi}\cdot t^{0.5}+C,$$

(4) $$q_{t}=\frac{1}{\beta }\ln\left(\alpha \beta t+1\right),$$

where $k_{pi}$ (mg/g min^0.5^) is the IPD rate constant; *t*^0.5^ is the square root of time; *C* (mg/g) is an intercept; *α* (mg/g min) is the initial adsorption rate; *β* (g/mg) is the desorption constant during each experiment.

The parameters obtained after fitting the experimental data to the PFO and PSO kinetic models are presented in Table 1. The fit plot of the models is shown in Figure 4. Both the pseudo-first and pseudo-second kinetic models describe accurately the kinetic process. However, the correlation coefficient R^2^ for the pseudo-first model was higher for all the initial concentrations of Mo(VI). Therefore, the pseudo-first kinetic model was found to be the best fitting model to describe the adsorption kinetics. The correlation coefficients of the Elovich equation were lower than those of the pseudo-second and pseudo-first order adsorption model, indicating that these models might not be suitable for the description of the adsorption mechanism. The calculated parameters of the model are presented in Table S1, and the fit plot of the model is shown in Figure S1.

In addition, to elucidate the rate-limiting step from a mechanistic point of view, the IPD model was applied to the kinetic experimental data. The plot of the dependence of the sorbed amount of Mo(VI), *q_t_* (mg/g), on the square root of the time, *t*^0.5^, showed that the extraction of Mo(VI) occurred in two stages until equilibrium was reached (Figure S2). The first rapid step can be associated with extracting Mo(VI) ions via sorption on the outer surface of the sorbent, followed by diffusion into the Al_2_O_3_ particle [30]. The second slow step demonstrates the phase of gradual adsorption and refers to the equilibrium stage. The slope of the straight line of these stages characterizes the velocity parameter *k_pi_* (mg/g min^0.5^) corresponding to intraparticle diffusion, the values of the intersection point C provide information on the thickness of the boundary layer, and the resistance to external mass transfer increases as the intersection point increases (Table S1). It should also be noted that the phase I graphs do not pass through the origin. This indicates that intraparticle diffusion participates in the adsorption process but is not the only mechanism controlling its rate.

#### 2.2.3. Adsorption Isotherms

The obtained data of the isotherms at different temperatures were calculated by Langmuir, Freundlich and Temkin models.

The Langmuir theory suggests that adsorption takes place on certain homogeneous surface areas of an adsorbent. Once all the areas are filled with a monolayer of adsorbed molecules, no extra adsorption or interaction between the adsorbed molecules can continue [30]. The nonlinear form of the Langmuir isotherm could be described as follows:

(5) $$q_{e}=q_{m}b_{l}c_{e}/\left(1+b_{L}c_{e}\right),$$

where $q_{m}$—maximum capacity of the monolayer (mg/g), and $b_{l}$—the adsorption coefficient (L/g).

The Freundlich isotherm is an empiric model, which not only concerns the covering of the surface of a sorbent by adsorbate molecules but also describes multilayer adsorption and takes into account interactions between the adsorbed molecules [30]. Mathematically, it can be presented as in Equation (6):

(6) $$q_{e}=K_{F}c_{e}{}^{1/n},$$

where $K_{F}$—the coefficient of distribution or adsorption coefficient (L/g).

Finally, according to Temkin’s isotherm model, it is presumed that the adsorption heat of all molecules decreases linearly with an increase in the degree of coverage of the adsorbent surface [39], as shown in Equation (7):

(7) $$q_{e}=\left(RT/b_{T}\right)\ln\left(Ac_{e}\right),$$

where $b_{T}$—the adsorption coefficient (J/mole); *R*—the universal gas constant of 8.314 J/(mol∙K); *A*—the constant, L/g; *T*—the absolute temperature (K).

**Figure 5:**
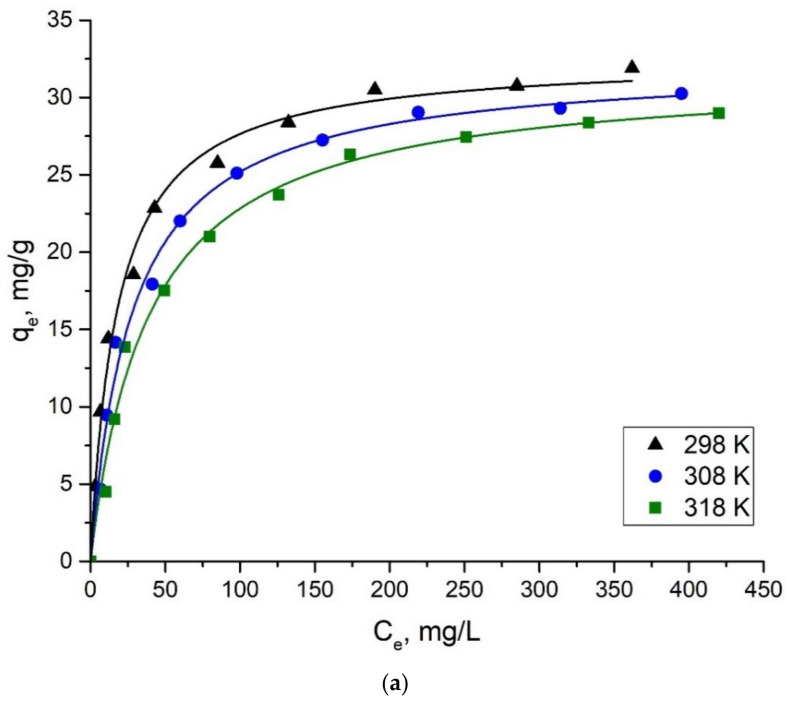

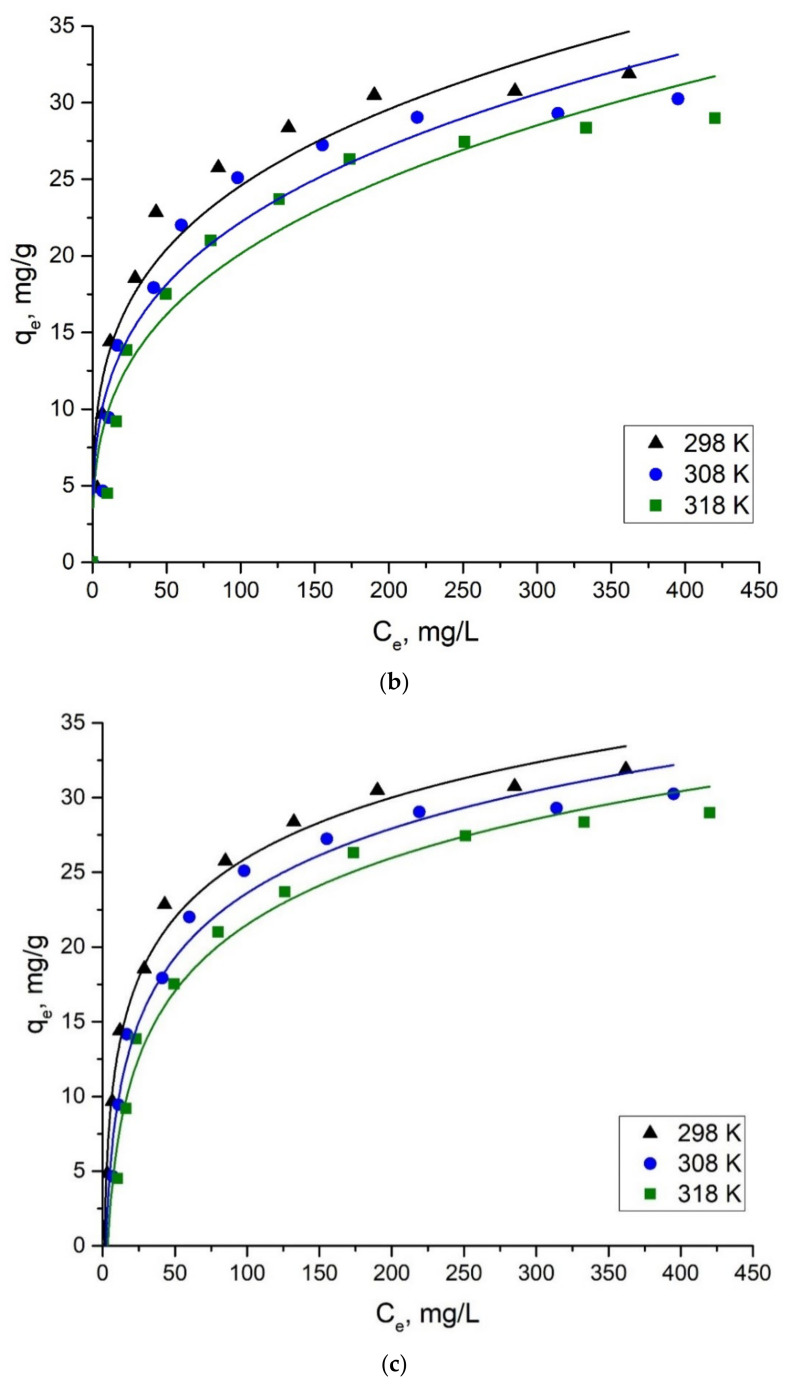
Plots of non-linear isotherm models for the adsorption of Mo(VI) ions on Al_2_O_3_ at different temperatures. (**a**) Langmuir model; (**b**) Freundlich model; (**c**) Temkin model.

Figure 5 shows the results of applying the isotherm models to the experimental data of the adsorption Mo(VI) ions on alumina at different temperatures. Experimental and calculated parameters for each isotherm model are given in Table 2.

It can be concluded from Table 2 that, according to the correlation coefficient (R^2^), the isotherm can be fitted with the Langmuir and Temkin isotherms with high precision, while Freundlich’s model is less accurate. The maximum adsorbing ability of alumina, its specific surface area and the time of the adsorption equilibrium onset of Mo(VI) compared to the results of other adsorption studies are shown in Table 3. It shows that commercial alumina demonstrates satisfactory adsorption capacity to molybdate ions in water solutions as well as a rapid onset of adsorption equilibrium.

#### 2.2.4. Thermodynamics Parameters

**Figure 6:**
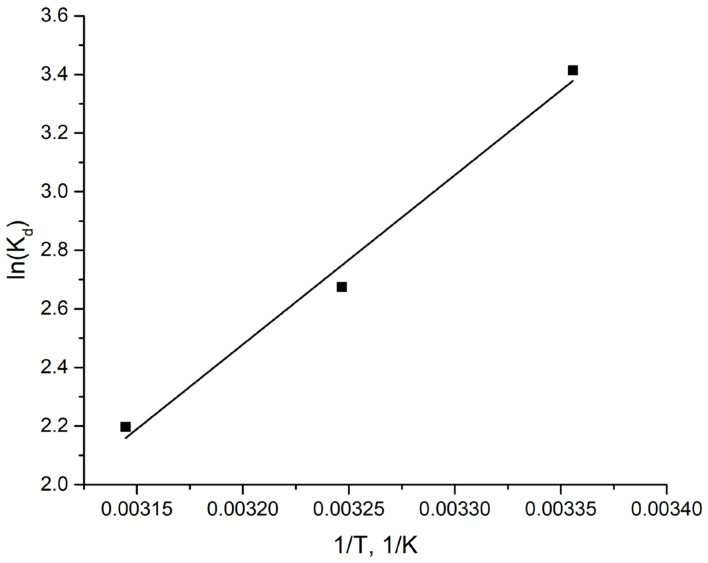
Linear plot of lnKd versus 1/T for the adsorption of Mo(VI) on Al_2_O_3_.

Gibbs free energy (ΔG, kJ/mol), enthalpy (ΔH, kJ/mol) and entropy (ΔS, kJ/mol∙K) are usually used to assess the trend of a chemical or physical process. These values have been pre-calculated at three different temperatures using Equations (8) and (9).

(8) $$\Delta G=-RTlnK_{d},$$

(9) $$lnK_{d}=-\frac{\Delta H}{RT}+\frac{\Delta S}{R},$$

where T (K) is the absolute temperature; R is the molar gas constant 8.3145 J/(mol/K); $K_{d}$ is the adsorption equilibrium constant. By plotting $lnK_{d}$ versus 1/T (Figure 6), the point of intersection with the X axis and the slope of the resulting line can be used to estimate ΔS and ΔH.

The obtained values of ΔG are negative and indicate that the adsorption of Mo(VI) on Al_2_O_3_ is spontaneous in the studied temperature range. A negative value of ΔH indicates that the adsorption process is exothermic (Table 4).

### 2.3. Desorption Studies

**Figure 7:**
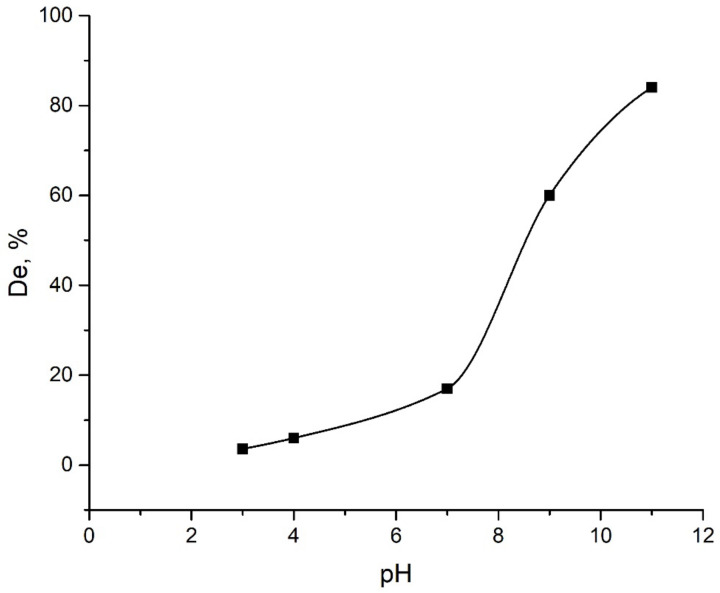
Desorption of Mo(VI) under different pH.

**Figure 8:**
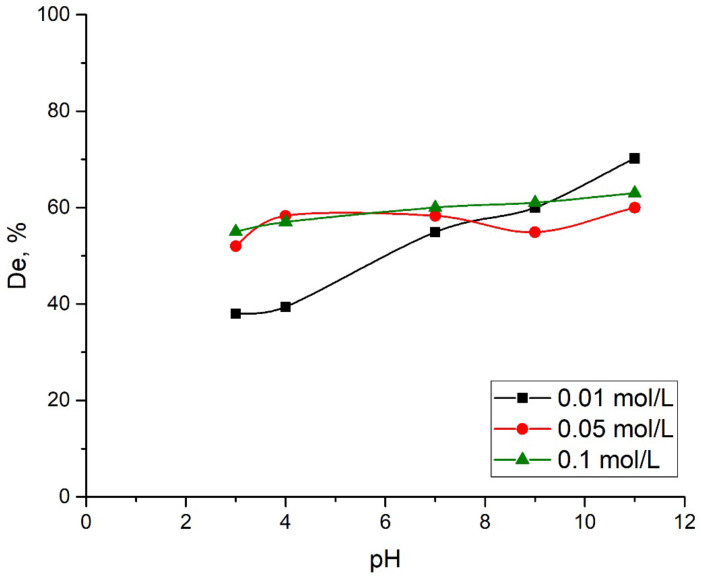
Desorption of Mo(VI) under different pH in Na_3_PO_4_ solution.

The desorption of the adsorbate from the surface of the adsorbent was studied to check the possibility of reusing the adsorbent. Two media were studied for the extraction of Mo(VI) from the surface of the adsorbent: an aqueous solution with different pH values (pH was controlled by adding 1 M NaOH or 1 M HCl) and sodium phosphate solutions with concentrations of 0.01, 0.05 and 0.1 mol/L with different pH values. The used adsorbent was placed in each of the media for 24 h with continuous mixing at a temperature of 298 K. The results of the desorption studies are presented in Figure 7 and Figure 8.

Figure 7 shows that the amount of desorption increases with the increasing pH of the solution. The high extraction of Mo(VI) when using 1 M NaOH is explained by the competition between the hydroxyl group and the Mo(VI) anion, which made it possible to easily separate the molybdenum anions bound to the aluminum oxide surface. The same was suggested in [45]. It should be noted that the use of an alkaline solution for regeneration is not always convenient due to the aggressiveness of the environment and the formation of side pollutants. Furthermore, it requires additional protective measures both during the regeneration and during the following washing of the regenerated adsorbent.

Based on the results provided in [28], the treatment of molybdenum-containing soils with a phosphate solution led to the desorption of Mo from them. Moreover, the intensity of the desorption did not depend on the pH value. This motivated us to study the influence of phosphate solutions of different concentrations and pH values on the desorption of Mo(VI) from the surface of the aluminum oxide. The results of the research are given in Figure 8.

Figure 8 shows that the treatment of spent alumina with a solution of Na_3_PO_4_ promotes the desorption of molybdate ions from its surface even at low pH values. It can be assumed that phosphate and molybdate ions compete with each other for the sorption centers of the aluminum oxide surface. In addition, according to the findings in [28], the adsorption of phosphate ions on the surface of the alumina while treating in Na_3_PO_4_ results in increasing negative charges on this surface. Since the molybdate ions are also negatively charged, the equilibrium shifts towards their desorption from the surface of aluminum oxide. It is likely that the desorption of Mo(VI) from the Al_2_O_3_ surface can be caused by both of these processes taking place simultaneously. However, additional physicochemical studies are needed for a more detailed description of the desorption mechanism.

Compared to an alkali solution, phosphate solutions are considered to be more environmentally friendly options. As a result of desorption, a slightly acidic or neutral solution containing molybdenum and phosphate ions can be obtained. Such a by-product can be used, e.g., in agriculture to enrich soils with a deficiency of molybdenum [46,47].

**Figure 9:**
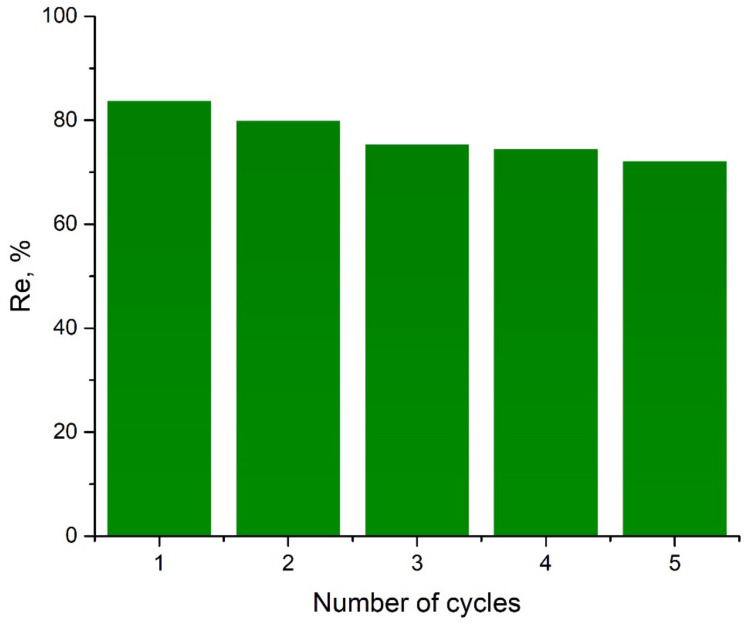
Effect of regeneration cycle on Mo(VI) adsorption on Al_2_O_3_.

To assess the possibility of multiple uses of aluminum oxide regenerated in a phosphate solution, several adsorption–desorption cycles were carried out using the same adsorbent sample. The desorption was performed using a Na_3_PO_4_ solution with a concentration of 0.1 mol/L. It was revealed that the adsorption capacity of Al_2_O_3_ for Mo(VI) lowers by ~18% after two cycles of using the adsorbent, which is considered to be an insignificant loss of activity. After the fifth adsorption–desorption cycle, the adsorption capacity of Al_2_O_3_ for Mo(VI) decreases by 28% (Figure 9). Therefore, the aluminum oxide used in the study demonstrates noticeable stability and the possibility of its reuse for the adsorption of Mo(VI) ions.

## 3. Materials and Methods

### 3.1. Chemicals and Reagents

All chemicals and reagents used in this work were of analytical grade (AR) and underwent no additional purification. Aluminum oxide (Al_2_O_3_) powder with a mean particle size of 16 µm was supplied from Component-reaktiv (Moscow, Russia). The molybdenum salt, Na_2_MoO_4_∙2H_2_O, was acquired from JSC LenReactiv (St. Petersburg, Russia)**.** NaOH, HCl, NaNO_3_ and HNO_3_ were obtained from Component-reaktiv (Moscow, Russia).

### 3.2. Adsorbent Characterization

The textural characteristics of the samples were studied using low-temperature nitrogen adsorption at 77 K on an Auto-sorb iQ gas sorption analyzer (Quantachrome Inst., Boynton Beach, FL, USA). The specific surface area was determined by the BET method. X-ray diffraction patterns (XRD) were acquired in the 2θ 15–120° range using an ARL X’tra spectrophotometer (Thermo Fisher Scientific, Ecublens, Switzerland). Qualitative X-ray phase analysis of the obtained spectra was carried out using the powder database ICDD PDF-2 (2008). Quantitative X-ray phase analysis according to the Rietveld method was carried out using the Siroquant Sietronics Pty Ltd. software (version 4) (Mitchell, Australia).

pH point of zero charge (pHpzc) was determined by potentiometric titration [48]. The titration was carried out in a cell thermostated at 25 °C and purged with purified nitrogen, using a Mettler Toledo Sevencompact pH meter (Mettler Toledo, LLC, Columbus, OH, USA). All solutions were prepared with bidistilled water and purged with purified argon for 30 min before the start of the experiment. A portion of oxide powder weighing 5 g was placed in 50 mL of NaNO_3_ for several hours until a constant pH value was established.

Before titration, a small volume of 1 M HNO_3_ solution was added to the suspension to protonate the surface areas. Then, the suspension was titrated by adding 0.05 mL of NaOH (0.1 M) accompanied by constant mixing. After each addition of NaOH, the pH value was recorded depending on the volume of titration solution added. The same treatment and procedure were used for a blank solution (0.03 M NaNO_3_). Equilibrium pH values were plotted versus the volume of added acid to obtain potentiometric curves. pH_PZC_ was identified as the point of intersection of the potentiometric curves with the titration curves of the blank solution.

### 3.3. Adsorption Studies

The adsorption of Mo(VI) on Al_2_O_3_ from aqueous solutions of a given concentration (100–1000 mg/L) was carried out in a thermostated cell with a reflux condenser with nonstop mixing with a magnetic stirrer (150 rpm) at 298 K, 308 K and 318 K. The pH of the solution was varied using 1 M of the HCl solution and 1 M of the NaOH solution. The adsorption kinetics were studied by tracking changes in the concentration of Mo(VI) at different times at pH 4 and with initial concentrations of molybdenum at 100, 300, 500, 700 and 1000 mg/L. To determine the concentration of the Mo(VI) in an aqueous solution, a thiocyanate photometric method [49] was used, which was carried out by absorption at a wavelength of 470 nm using the Hitachi U-1900 spectrophotometer (Hitachi Ltd., Tokyo, Japan).

Equation (8) was used to estimate the amount of ions of metal adsorbed per 1 g of Al_2_O_3_ (mg/g):

(10) $$q_{e}=\frac{\left(C_{0}-C_{e}\right)\cdot V}{m},$$

where $C_{0}$ and $C_{e}$—initial and equilibrium concentration of Mo(VI) ions, mg/L, respectively; V—volume of the solution, L; m—the mass of the adsorbent, g.

The degree of the removal of Mo(VI), represented as $Adsorbed\;\left(Mo\right)\;$(%), from solutions for different pH was calculated by Equation (11):

(11) $$Adsorbed\;\left(Mo\right)=\frac{\left(C_{0}-C_{e}\right)}{C_{0}}\cdot 100\%.$$

### 3.4. Desorption Studies

During the desorption studies, the adsorbent used for adsorption was separated by filtering, washed with deionized water to remove residual Mo and dried at room temperature. The desorption experiments were carried out by adding to a conical flask 1 g of spent adsorbent and 50 mL of deionized water or Na_3_PO_4_ solution. Then, HCl or NaOH (1 M) was added to achieve the necessary initial pH. The initial concentration of a Mo(VI) solution, which was used for the adsorbing saturation of Al_2_O_3_, was 100 mg/L.

The efficiency of desorption De (%) is the ratio of the amount of the compound desorbed from the gram of the adsorbent during the regeneration process, $q_{des}$ (mg/g), and the initial adsorption ability of the sorbent to the targeted compound, $q_{e}$ (mg/g):

(12) $$De\;\left(Mo\right)=\frac{q_{des}}{q_{e}}\cdot 100\%.$$

To evaluate the effectiveness of regeneration of aluminum oxide, the adsorption–desorption cycle was carried out five times. The effectiveness of regeneration Re (%), is the ratio of the adsorbing ability of the regenerated sorbent $q_{2}$ (mg/h) and its initial adsorbing capacity by the desired compound $q_{e}$ (mg/g) [50]. The effectiveness of regeneration was calculated according to Equation (13):

(13) $$Re=\frac{q_{2}}{q_{e}}\cdot 100\%.$$

## 4. Conclusions

Aluminum oxide is an inexpensive and affordable material. Within this study, aluminum oxide proved to be an efficient adsorbent for extracting the molybdate ions from aqueous solutions. The optimal conditions for adsorbing Mo(IV) on Al_2_O_3_ were found to be a media temperature of 298 K and a pH ranging from 4 to 7. Under optimal conditions, the adsorption process can be described by the pseudo-first order kinetics model, and experimental data can be fitted by the Langmuir isotherm model. The good compliance of the experimental data on the adsorption of the Langmuir model indicates that the adsorption of the Mo(VI) includes mono-layered adsorption on the Al_2_O_3_ surface. According to the Langmuir’s isotherm, the maximum adsorption ability of Al_2_O_3_ in Mo(VI) is 32.61 mg/g at 298 K. Thermodynamic calculations show that Mo(VI) adsorption is spontaneous and exothermic. This work proves that the treatment of the adsorbent with the phosphate solution effectively adsorbs molybdate onto the alumina surface. Furthermore, aluminum oxide can be reused at least twice without a significant loss of its adsorption ability. The third, fourth and fifth regeneration cycles decrease the sorption capacity of the alumina from 80% to 70%. It can still be used for adsorption as an additive to a new portion of the adsorbent.

## Figures and Tables

**Table 1:** Kinetic models for the removal of molybdate using Al_2_O_3_.

C_0_ Mo (VI), mg/L	Pseudo-First Order			Pseudo-Second Order			*q_e_* Experimental, mg/g
	*k*_1_	*q_e_*_1_, mg/g	R^2^	*k*_2_	*q_e_*_2_, mg/g	R^2^	
100	0.186	4.694	0.977	0.099	4.722	0.921	4.841
300	0.048	14.205	0.998	0.217	13.991	0.992	14.294
500	0.048	22.812	0.986	0.003	24.185	0.953	22.856
700	0.036	28.423	0.996	0.002	30.456	0.972	28.385
1000	0.037	31.187	0.996	0.002	33.431	0.976	31.9

**Table 2:** Isotherm parameters for Mo(VI) adsorption on Al_2_O_3_.

Model Parameters		Temperature, K		
		298	308	318
Langmuir	*q_m_*, mg/g	32.61	32.246	31.662
	*b_l_*, L/g	0.056	0.036	0.026
	R^2^	0.994	0.991	0.993
Freundlich	*K_F_*, mg/g	7.189	5.794	4.681
	1/*n*	0.267	0.292	0.317
	R^2^	0.923	0.902	0.912
Temkin	*b_T_*, J/mol	427.204	410.582	410.835
	*A*, L/g	0.883	0.441	0.283
	R^2^	0.991	0.982	0.985

**Table 3:** Comparison of capacity values for Mo(VI) metal ions sorbed by different adsorbents.

Adsorbent	*q_e_*, mg/g	S_BET,_ m^2^/g	Equilibrium Time, min	Ref.
γ-Al_2_O_3_	31	100	-	[40]
Magnetic Cr-ferrite	26.8	-	180	[30]
Mo(VI) ion-imprinted polymer	126.06	307.32	10	[41]
chitosan sorbent	124.34	-	15	[42]
Modified drinking water treatment residues	39.52	36.73	-	[23]
Activated carbon	16.54	-	30	[43]
Multiwalled carbon nanotubes	18.4	167	360	[44]
Commercial Al_2_O_3_	31.9	65.5	100	This work

**Table 4:** Thermodynamic parameters for Mo(VI) adsorption on Al_2_O_3_ at C_0_ = 100 mg/L.

*T*, K	Δ*G*, kJ/mol	Δ*H*, kJ/mol	Δ*S*, kJ/mol∙K
298	−8.41	−48.044	−0.133
308	−7.08		
318	−5.75		

## Data Availability

The data described in this article are openly available in previous works.

## Supplementary Materials

The following supporting information can be downloaded at: https://www.mdpi.com/article/10.3390/ijms24108700/s1.
