# Supplementary material for: Adsorption Removal of Mo(VI) from an Aqueous Solution by Alumina with the Subsequent Regeneration of the Adsorbent
Source: Int J Mol Sci. 2023 May 12;24(10):8700. doi: 10.3390/ijms24108700 (PMC10218439; doi:10.3390/ijms24108700)
Supplement: Supplementary file 1 [file ijms-24-08700-s001.zip › ijms-2331217-supplementary.pdf]

Supplementary Materials

Alexandra Yu. Kurmysheva [1,*], Marina D. Vedenyapina [2], Stanislav A. Kulaishin [2], Pavel Podrabinnik [1,3], Nestor Washington Solís Pinargote [1], Anton Smirnov [1], Alexander S. Metel [3], José F. Bartolomé [4] and Sergey N. Grigoriev [1,3]

1 Laboratory of Electric Current Assisted Sintering Technologies, Moscow State University of Technology "STANKIN", Vadkovsky per. 1, 127055 Moscow, Russia; p.podrabinnik@stankin.ru (P.P.); nw.solis@stankin.ru (N.W.S.P.); a.smirnov@stankin.ru (A.S.); s.grigoriev@stankin.ru (S.N.G.)
2 N. D. Zelinsky Institute of Organic Chemistry, Russian Academy of Sciences, Leninsky Prospect 47,
119991 Moscow, Russia; mvedenyapina@yandex.ru (M.D.V.); s.kulaishin@mail.ru (S.A.K.)
3 Department of High-Efficiency Machining Technologies, Moscow State University of Technology, "STANKIN", Vadkovsky per. 1, 127055 Moscow, Russia; a.metel@stankin.ru
4 Instituto de Ciencia de Materiales de Madrid (ICMM), Consejo Superior de Investigaciones Científicas (CSIC), c/Sor Juna Inés de la Cruz, 3, Cantoblanco, 28049 Madrid, Spain; jbartolo@icmm.csic.es
* Correspondence: a.kyrmisheva@stankin.ru

Table S1. Kinetic models for the removal of molybdate using Al_2O_3.

C_0 Mo	IPD						Elovich equation		
(VI), mg/l	k_{p1}, mg/g min$^{1/2}$	C_1, mg/g	R^2	k_{p2}, mg/g min$^{1/2}$	C_2, mg/g	R^2	α, mg/(g·min)	β, g/mg	R^2
**100**	1.009	0.208	0.931	0.04	4.125	0.784	1.143×10^9	5.96	0.993
300	3.106	0.629	0.933	0.077	12.89	0.722	2.186×10^{15}	2.985	0.997
500	2.566	0.993	0.903	0.07	21.524	0.857	42.437	0.378	0.838
700	2.75	1.204	0.943	0.054	27.429	0.552	15.24	0.255	0.86
1000	2.967	1.673	0.932	0.149	28.637	0.774	17.51	0.234	0.868

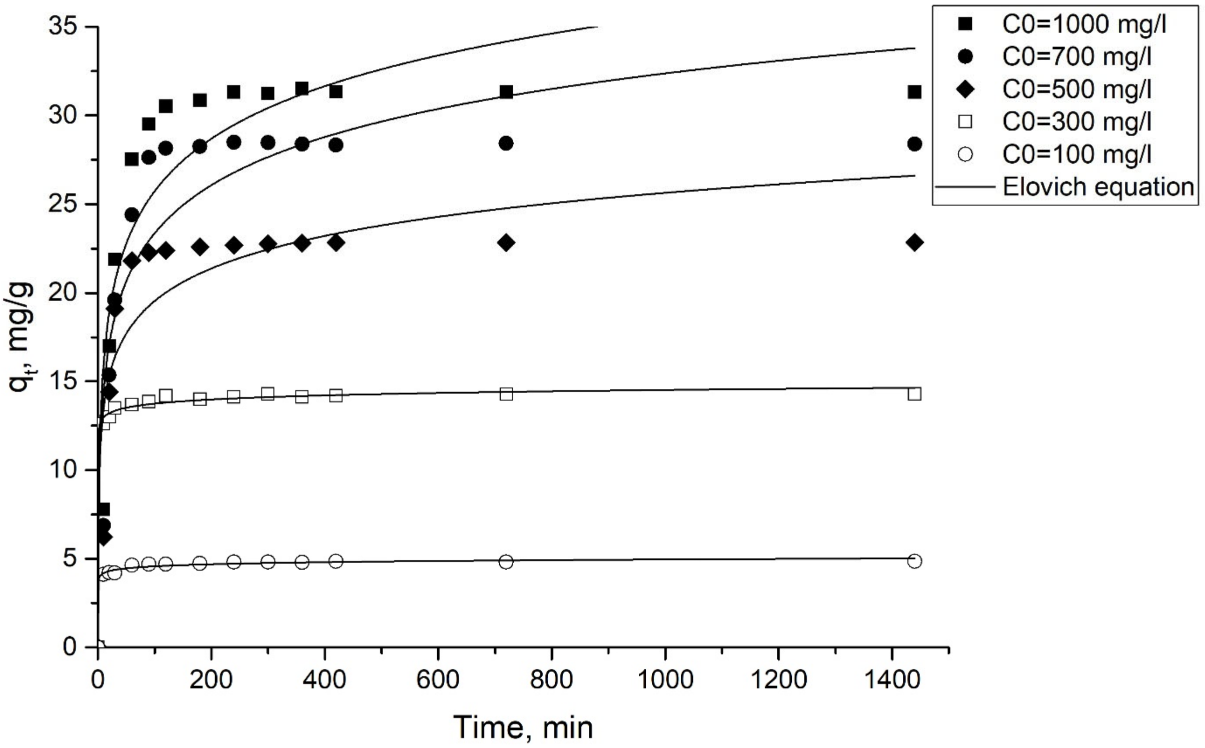

Figure S1. Elovich kinetic model for the adsorption of Mo(VI) on the Al_2O_3.

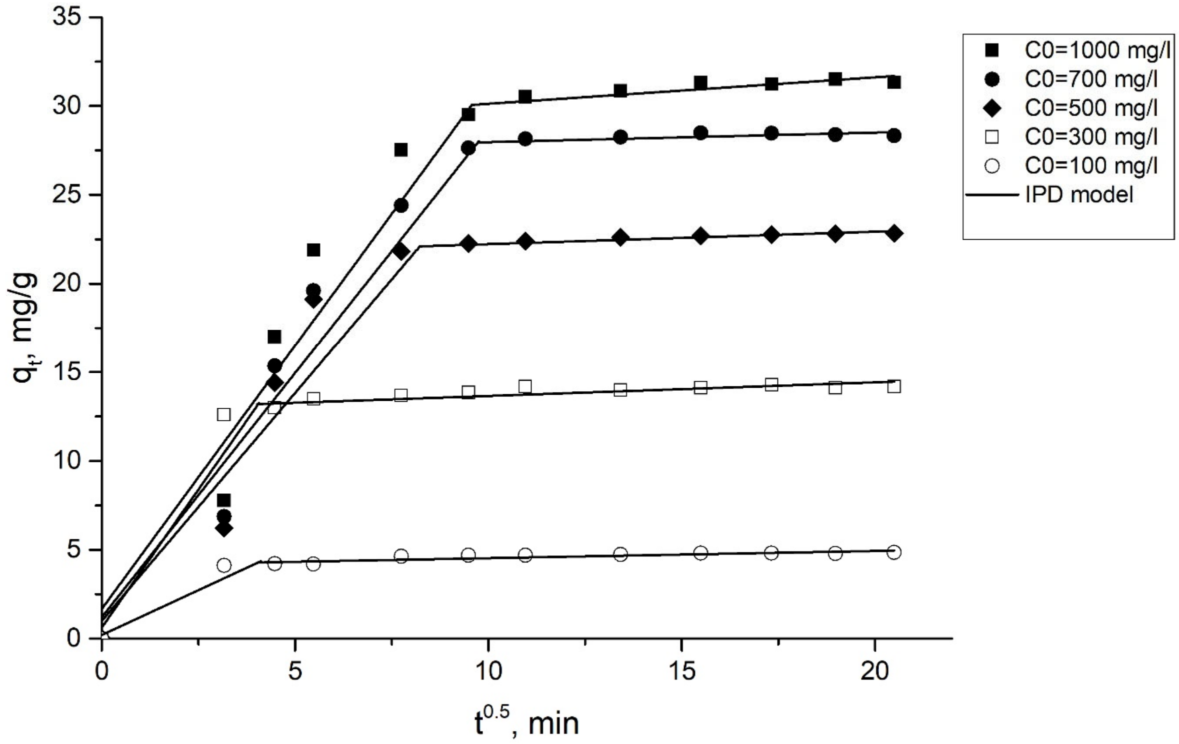

Figure S2. Intra-particle diffusion kinetic model for the adsorption of Mo(VI) on the Al_2O_3. Time up to 720 minutes.
